# Proteinaceous secretion of bioadhesive produced during crawling and settlement of *Crassostrea gigas* larvae

**DOI:** 10.1038/s41598-018-33720-4

**Published:** 2018-10-17

**Authors:** Valentin Foulon, Sébastien Artigaud, Manon Buscaglia, Benoit Bernay, Caroline Fabioux, Bruno Petton, Philippe Elies, Kada Boukerma, Claire Hellio, Fabienne Guérard, Pierre Boudry

**Affiliations:** 1 Laboratoire des Sciences de l’Environnement Marin (LEMAR), UMR 6539 CNRS/UBO/IRD/Ifremer, Institut Universitaire Européen de la Mer, Technopole Brest-Iroise, Rue Dumont d’Urville, 29280 Plouzané́, France; 2 Plateforme Proteogen SF ICORE, Université de Caen Basse-Normandie, 14032 Caen Cedex, France; 3 Ifremer, Laboratoire des Sciences de l’Environnement Marin, UMR 6539 CNRS/UBO/IRD/Ifremer, Centre Bretagne, 29280 Plouzané, France; 4 Plateforme d’Imagerie et de Mesures en Microscopie, Université de Bretagne Occidentale, 29200 Brest, France; 5 Ifremer, Laboratoire Détection, Capteurs et Mesures (LDCM), Centre Bretagne, 29280 Plouzané, France

**Keywords:** Proteins, Proteomics

## Abstract

Bioadhesion of marine organisms has been intensively studied over the last decade because of their ability to attach in various wet environmental conditions and the potential this offers for biotechnology applications. Many marine mollusc species are characterized by a two-phase life history: pelagic larvae settle prior to metamorphosis to a benthic stage. The oyster *Crassostrea gigas* has been extensively studied for its economic and ecological importance. However, the bioadhesive produced by ready to settle larvae of this species has been little studied. The pediveliger stage of oysters is characterized by the genesis of a specific organ essential for adhesion, the foot. Our scanning electron microscopy and histology analysis revealed that in *C. gigas* the adhesive is produced by several foot glands. This adhesive is composed of numerous fibres of differing structure, suggesting differences in chemical composition and function. Fourier transformed infrared spectroscopy indicated a mainly proteinaceous composition. Proteomic analysis of footprints was able to identify 42 proteins, among which, one uncharacterized protein was selected on the basis of its pediveliger transcriptome specificity and then located by mRNA *in situ* hybridization, revealing its potential role during substrate exploration before oyster larva settlement.

## Introduction

In aquatic ecosystems, adhesion of organisms is a complex phenomenon due to the high diversity of substrates and variability of environments. Biofouling, which can be defined as the adhesion and subsequent growth of organisms on a substrate has been extensively described in marine environments^1^. It can be observed at every depth, from the surface to the bottom of the ocean, on floating or fixed substrates, which may be organic or inorganic, biotic (alive or dead) or abiotic, soft or hard. Settlement on a substrate is a strategy used by aquatic organisms to ensure survival, feeding, a high rate of reproduction, and sometimes metamorphosis; some species show gregarious traits and can induce settlement of conspecifics by chemical signalling^2,3^. Thus, in order to adhere to a substrate, organisms can use mechanical and/or chemical strategies. The latter imply the synthesis and use of bioadhesive polymers, which are usually mostly composed of a mix of proteins and polysaccharides^4,5^. However, inorganic components can sometimes predominate, e.g. representing 86% of adhesive from reef building oysters^6,7^. Lipids can also be produced, as observed in barnacle larvae, and play a role in the conditioning of the substrate prior to adhesive secretion^8^. Mechanical and chemical strategies can generate reversible or irreversible adhesion. Irreversible adhesion occurs in many benthic organisms that are firmly stuck to substrates, such as macroalgae^9^ or some bivalves like mussels^10^ or oysters^11^.

In recent decades, increasing research has been dedicated to marine bioadhesives^12^. One of the main characteristics of marine bioadhesives is their ability to polymerize very quickly in water (within a few minutes), and with a large scale of strengths^13^. Biomimetic formulations inspired by marine bioadhesives could offer a higher strength than the best synthetic glue actually on the market^14^. Moreover, most synthetic adhesives are formulated with toxic solvents, while the formulation of biomimetic bioadhesives can be achieved without solvents and can therefore be biocompatible, allowing biomedical applications^15^. Mussel byssus is the best known marine bioadhesive, having been studied for over forty years^16^. Byssus, specifically produced by the foot of the mussel, is composed of a variety of mussel foot proteins showing post-translational hydroxylation of tyrosine with 3,4-dihydroxyphenylalanine (DOPA) motifs^16,17^. Intensive research efforts on the characterization of byssus protein resulted in biotechnological applications of these proteins^14,18,19^. However, many other marine organisms that strongly adhere to their substrate by bioadhesives remain to be investigated. Most bivalves secrete adhesive during the settlement stage of their life cycle, which marks the transition between pelagic larval stage and benthic adult stage by metamorphosis^20^. Oysters, which are permanently fixed on their substrate at the adult stage, settle definitively at the larval stage prior to metamorphosis. Living in the intertidal zone, Pacific oysters *Crassostrea gigas* are exposed to harsh conditions and could potentially produce bioadhesives with outstanding properties. *C. gigas* is among the most studied marine organisms because of its economic and environmental importance. Its life cycle has been extensively described and artificial reproduction is fully mastered, allowing mass hatchery production of juveniles. *C. gigas* larval development commonly lasts about 15 days at 25 °C, depending on temperature and food availability.

At this stage, pediveliger larvae, with shell lengths of 280 to 300 µm, are characterized by the presence of a foot: an organ specific to the settlement phase in oysters. When larvae stop swimming and sink, the foot explores the substrate by crawling for a few minutes. Finally, at the end of the crawling phase, the foot secretes the final adhesive for definitive attachment. Metamorphosis of *C. gigas* larvae occurs a few hours later and the foot is totally resorbed after this stage^11^. In other bivalve species that are not definitively fixed to their substrate, the foot is not resorbed during metamorphosis and can develop to become a highly specific organ, like in mussels.

One of the main challenges of studying adhesives from larvae is to obtain sufficient material for further analysis. Indeed, previous research estimated the volume of oyster adhesive secretion at a few hundred picolitres^21^. Since this first investigation on European flat oyster *O. edulis*^22^, 45 years ago, only a few papers have been published on oyster larval settlement and their adhesive composition. This knowledge would be useful for the optimization of larval settlement under natural or hatchery conditions of this important aquaculture organism and might lead to the development of novel biomimetic bioadhesives.

In our research project, we investigated the adhesion process and adhesive composition of *C. gigas* larvae. Foot structure was described using histology and scanning electron microscopy (SEM) techniques. The adhesive was first examined through microscopy observations, spectrometry and proteomic analysis. Our proteomic analysis of *C. gigas* larvae was based on genome and transcriptome data from Zhang *et al*.^23^. For each detected protein, corresponding gene expression level was analysed for each developmental stage using transcriptomic databases^23^. One candidate gene, selected based on its specific expression profile at pediveliger stage, has been deeply studied by *in situ* hybridization.

## Results

### Foot anatomy and adhesive structure

**Figure 1:**
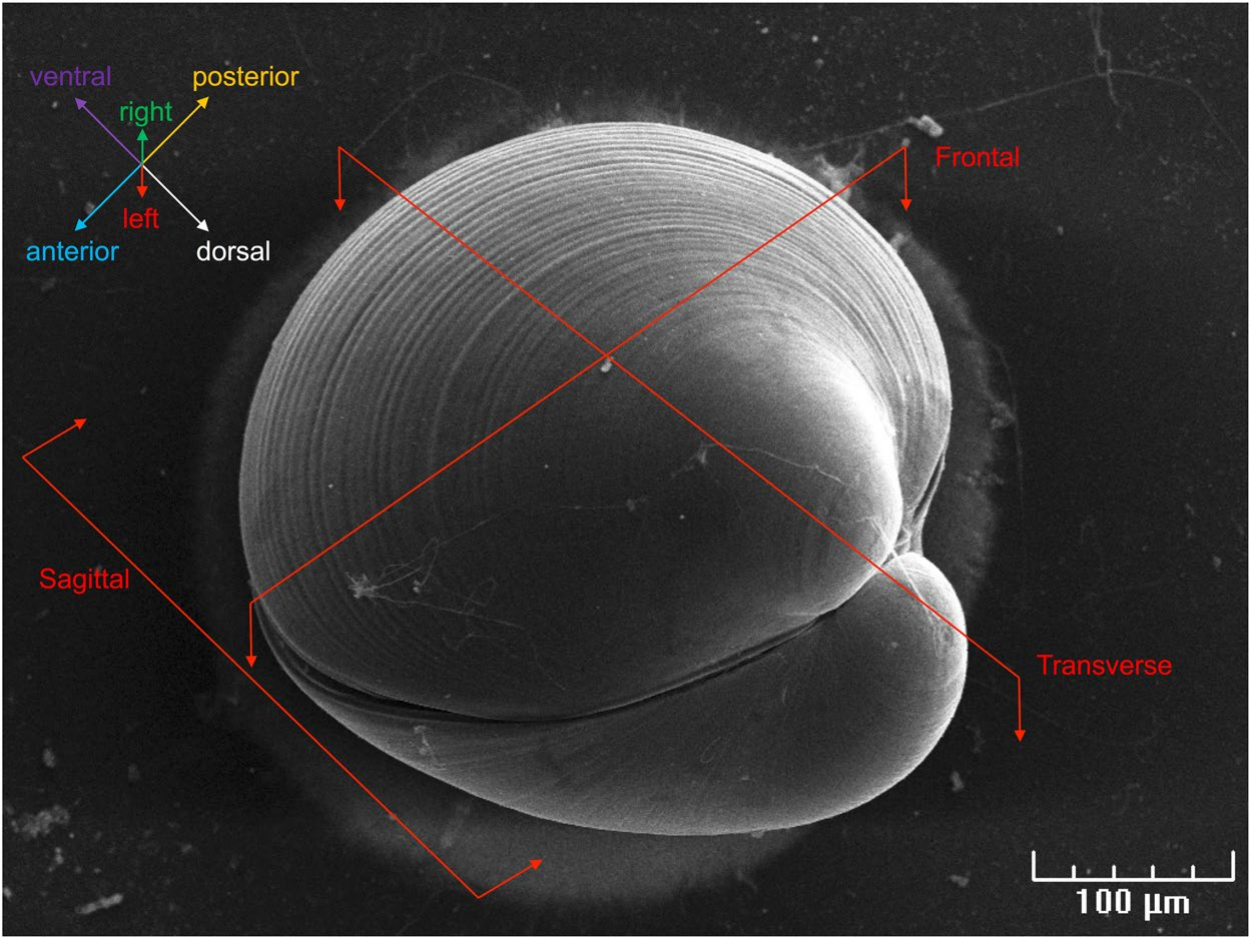
Scanning electron microscopy observation of a settled *Crassostrea gigas* pediveliger larva. Three dimensional animal orientation and planes of sections are indicated.

**Figure 2:**
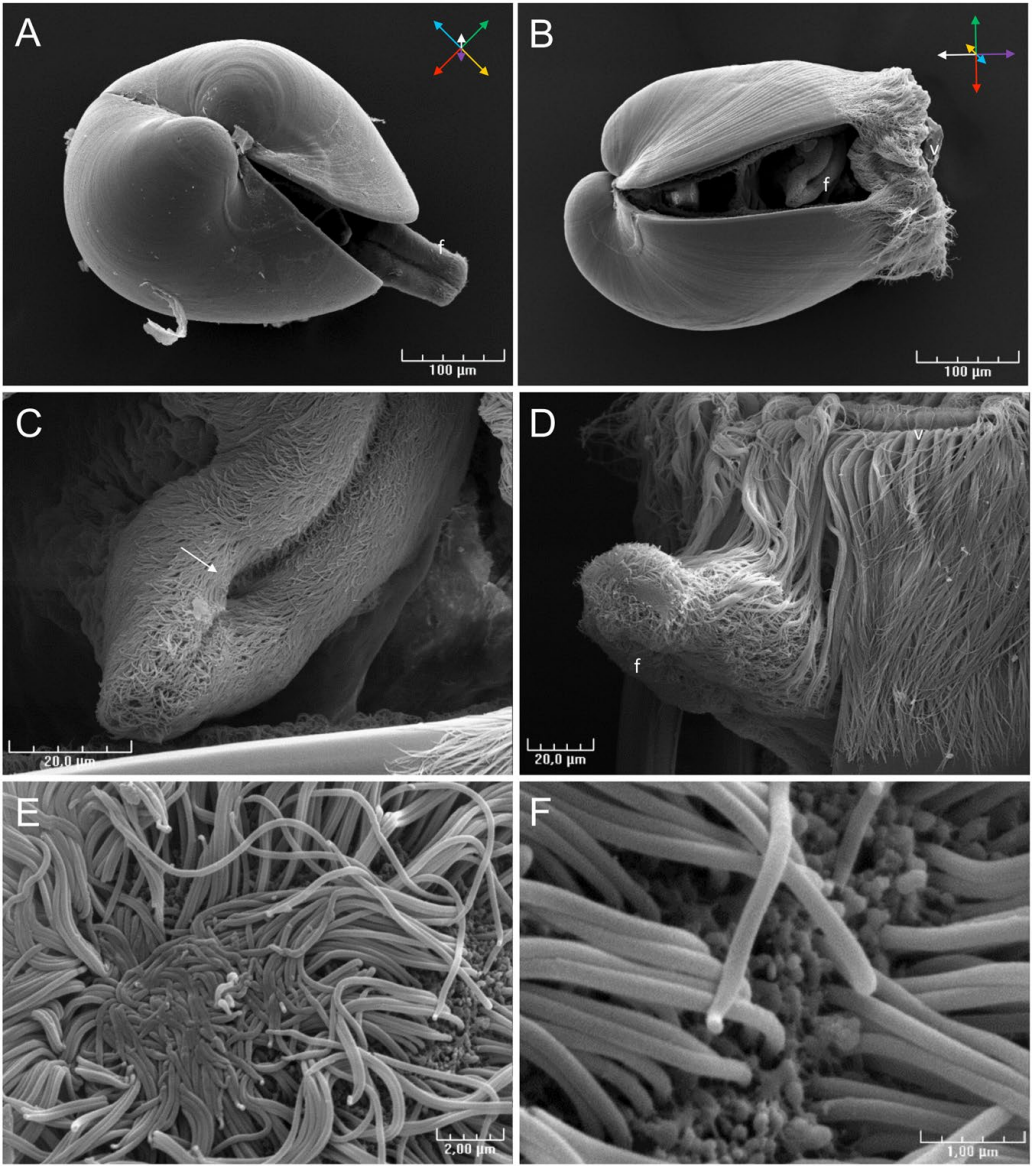
Scanning electron microscopy observation of a *Crassostrea gigas* pediveliger larva. (**A**) Dorsal view, the foot is extended out from the larva. (**B**) Posterior view, the foot is retracted behind the velum. (**C**) The byssus duct (arrow) is located next to the heel of the foot. (**D**,**E**) Observation of the tip of the foot of a crawling larva showing a flattened cilia zone. (**F**) Vesicles observed on the epithelium surface of the tip of the foot, at the base of cilia. f: foot, v: velum.

Figure 1 depicts a settled *C. gigas* pediveliger larva observed by scanning electron microscopy (SEM) and indicates the anatomical planes of sections used in histology (see below). The foot, approximatively 140 µm long and 40 µm wide, is located in the posterior part of the larva (Fig. 2). SEM observations show the longitudinal symmetry of the foot (Fig. 2A–C). Cilia are present all around the foot from the tip to the heel, but are absent at the base of the foot, behind the heel (Fig. 2B,C). The byssus duct was observed at the anterior face of the foot in front of the heel (Fig. 2C). The tip of the foot of crawling pediveliger larvae has a zone where cilia are stuck together (Fig. 2D,E). On the tip of the foot, at the base of the cilia, numerous vesicles of 50 to 150 nm can be observed on the surface of the epidermis (Fig. 2F).

**Figure 3:**
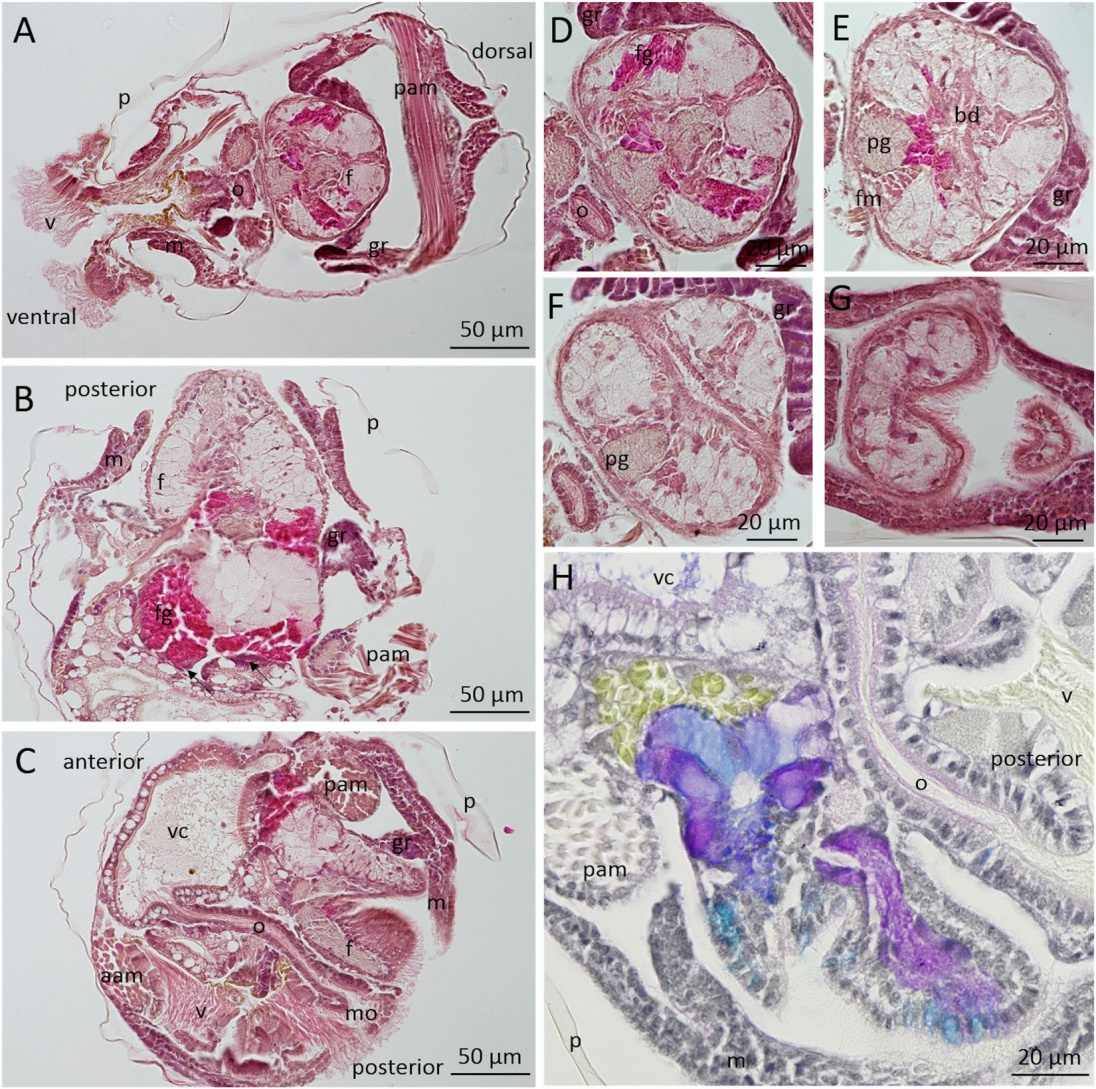
Histological observation of *Crassostrea gigas* pediveliger larvae stained with hematoxylin and eosin (**A**–**G**) and multichrome technic (Alcian Blue, Periodic Acid–Schiff’s, Haematoxylin, Picric Acid) (**H**). A: Transverse, B: Frontal, C and H: Sagittal sections of the whole larvae. (**D**–**G**) show serial transverse sections of a foot. aam: anterior adductor muscle, bd: byssus duct, f: foot, fg: foot gland, fm: foot muscle, gr: gill rudiment, m: mantle, mo: mouth, o- oesophagus, p: periostracum, pam: posterior adductor muscle, pg: pedal ganglia, v: velum, vc: visceral cavity, arrow: secreting cells from the foot gland.

An anatomical description of pediveliger larvae (Fig. 3A–C) and more specifically of the foot (Fig. 3D–G) was made from histological sections. The three anatomical section planes indicated in Fig. 1 are presented in Fig. 3: a transverse section (Fig. 3A) making it possible to observe the longitudinal bilateral symmetry, a frontal section (Fig. 3B), and a sagittal section (Fig. 3C). The mantle (m), observed on both section planes, produces the periostracum (p) from the periostracal groove. The periostracum is a protective and structural organic part of the shell, remaining after decalcification. The velum (v), shown deployed in Figs 2B,D and 3A and retracted in Fig. 3C is a ciliated organ used by larvae to swim and to collect food particles. The food particles are then ingested by the mouth (mo), a ciliated orifice connected to the visceral cavity (vc) by the oesophagus (o) (Fig. 3C). Adductor muscles (posterior and anterior) located on both sides of the visceral cavity (Fig. 3C) link the two shell valves (Fig. 3A). The foot is surrounded by rudimentary gills (Fig. 3A) and is linked at the base to the posterior adductor muscle (Fig. 3B,C).

Serial transversal sections of the foot were performed from the anterior (heel) to the posterior (tip) (Fig. 3D–G). The main structures of the foot observed on the slides are: a ciliated epithelium that can be observed on the external part of the foot (Fig. 3C,F,G); circular nerve structures corresponding to pedal ganglia (pg) of approximatively 20 µm in diameter, located on the anterior part of the foot (Fig. 3E,F); muscular fibres observed around the pedal ganglia and progressing along the foot (Fig. 3D,E); conjunctive-like tissue; and the principal foot adhesive gland (fg) (Fig. 3A–E). This gland contains strongly eosinophilic granules (in pink), indicating a proteinaceous composition, and is located at the base of the foot (Fig. 3B). In Fig. 3H, the positive reaction to picric acid in yellow of this granules confirmed the protein content. Massive secreting cells, stained in purple, are located at the base of this gland between the foot and the visceral cavity (Fig. 3B – arrow). This main gland is divided into two parts (Fig. 3D), which lead to the byssus duct (Fig. 3E). Live light microscopy observations of cementing larvae indicate the central role of this gland during adhesion, releasing the majority of the final adhesive (see Supplementary Video 1). Immediately after secretion, adhesive was observed to have polymerized and settled larvae by their left valve. In the Fig. 3H, five other groups of cells stained differently by the multichrome technic were described. By comparisons, nomenclature used in *O. edulis* was used:

(i) a row of turquoise blue cells distributed in the epithelium of the underside of the foot were positive to alcian blue staining indicating a high acid polysaccharides content (Gland A).
(ii) cells with purple staining (neutral polysaccharides), browse through the length of the foot, from the pedal ganglion to the tip, where they appeared to lead to a row of 3 to 4 cells (Gland B).
(iii) the byssus duct of *C. gigas* pediveliger larvae is surrounded by three cell types, called glands D. Massive cells, rich in acidic polysaccharides, were observed attached to the main gland of adhesive. These cells, about 20 µm by 5 µm in size, appeared to lead directly into the byssus duct. Other cells with the same morphology, but stained in purple with a dark blue perimeter were observed on either side of the byssus duct. Finally, small dark blue cells (1 to 2 µm in diameter) were observed on the lower part of the byssus duct, near the heel of the foot.

**Figure 4:**
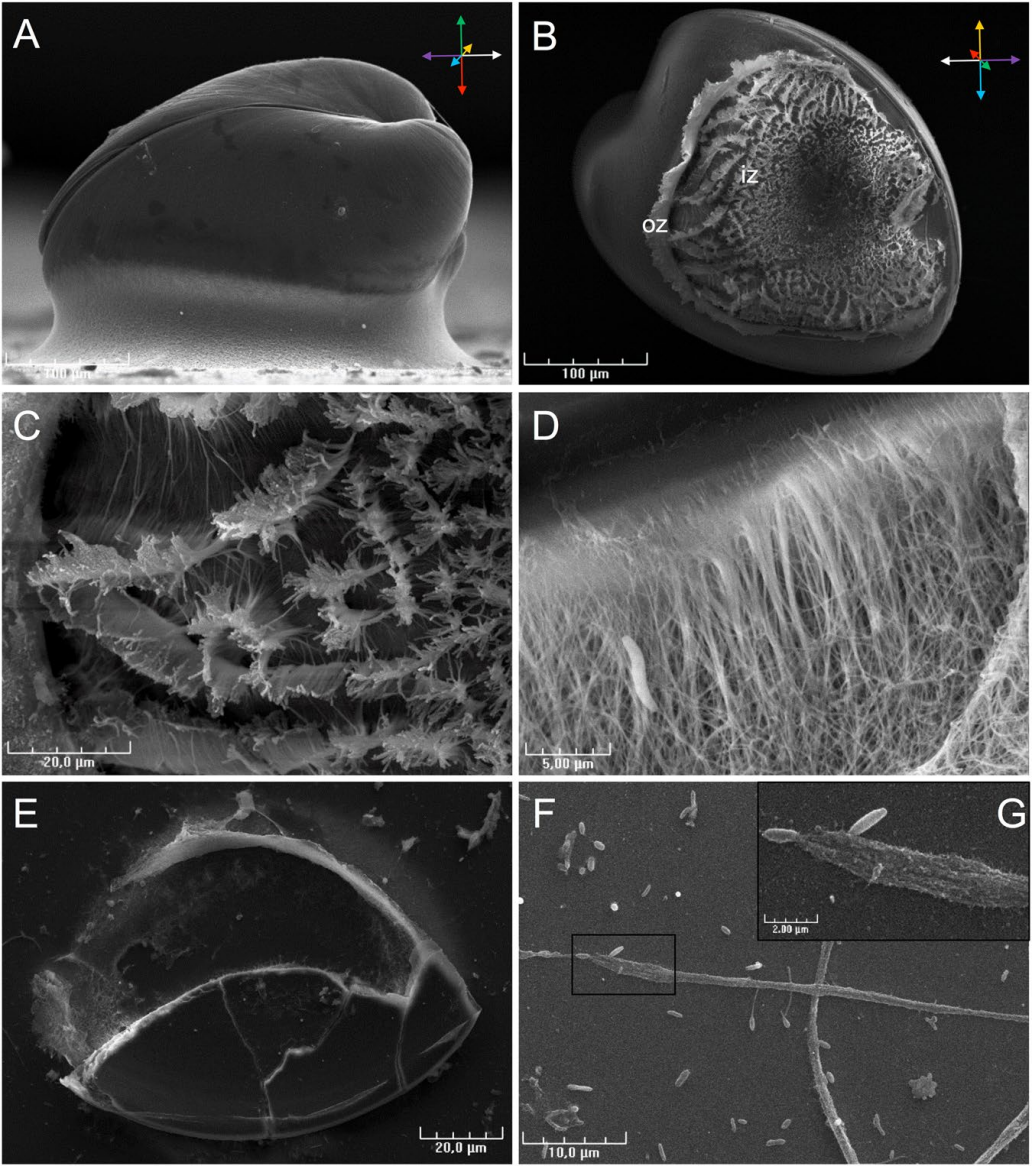
Scanning electron microscopy (SEM) observations of settled *Crassostrea gigas* pediveliger larvae. (**A**) Lateral view of a settled larva showing adhesive sealant between the left shell and the substrate. (**B**) The larva was removed from the substrate to visualize the adhesive structure. Two types of fibre are observed, composing the outer zone (oz) and inner zone (in). (**C**) In the inner zone, fibre shape is typical of the substrate roughness. (**D**) Fibres of the outer zone in contact with the shell (at the top) are larger and converge in a homogenous substance at the edge of the mantle. Bacteria were observed stuck in the fibres of the outer zone. (**E**) Adhesive footprint. (**F**) Byssus filament was observed around settled larvae. (**G**) High magnification indicates the presence of microfibres that stuck the byssus filament to the substrate.

SEM observations clearly revealed that the adhesive forms a perfect seal between the left shell of the larva and the substrate (Fig. 4A). SEM analyses also highlighted the fibrous structure of *C. gigas* adhesive (Fig. 4B–D). Two main zones could be differentiated in the adhesive, with variations in fibrous structure: the outer zone (oz), composed of tight filaments like a protective collar, and the inner zone (iz), composed of slacker vertically ordered fibres (Fig. 4B,C). In the outer zone, near the shell, fibres coalesce into larger ones and form a packed structure (Fig. 4D). Bacteria were present in the adhesive and around settled larvae (Fig. 4D,F). After removing larvae from the substrate with micro-forceps, a part of the adhesive remained present with shell debris (Fig. 4E). Around settled larvae, filaments were observed with a diameter of 1 to 2 µm. Higher magnification of these filaments revealed nanostructures which stuck them to the substrate (Fig. 4F,G).

### Adhesive composition

**Figure 5:**
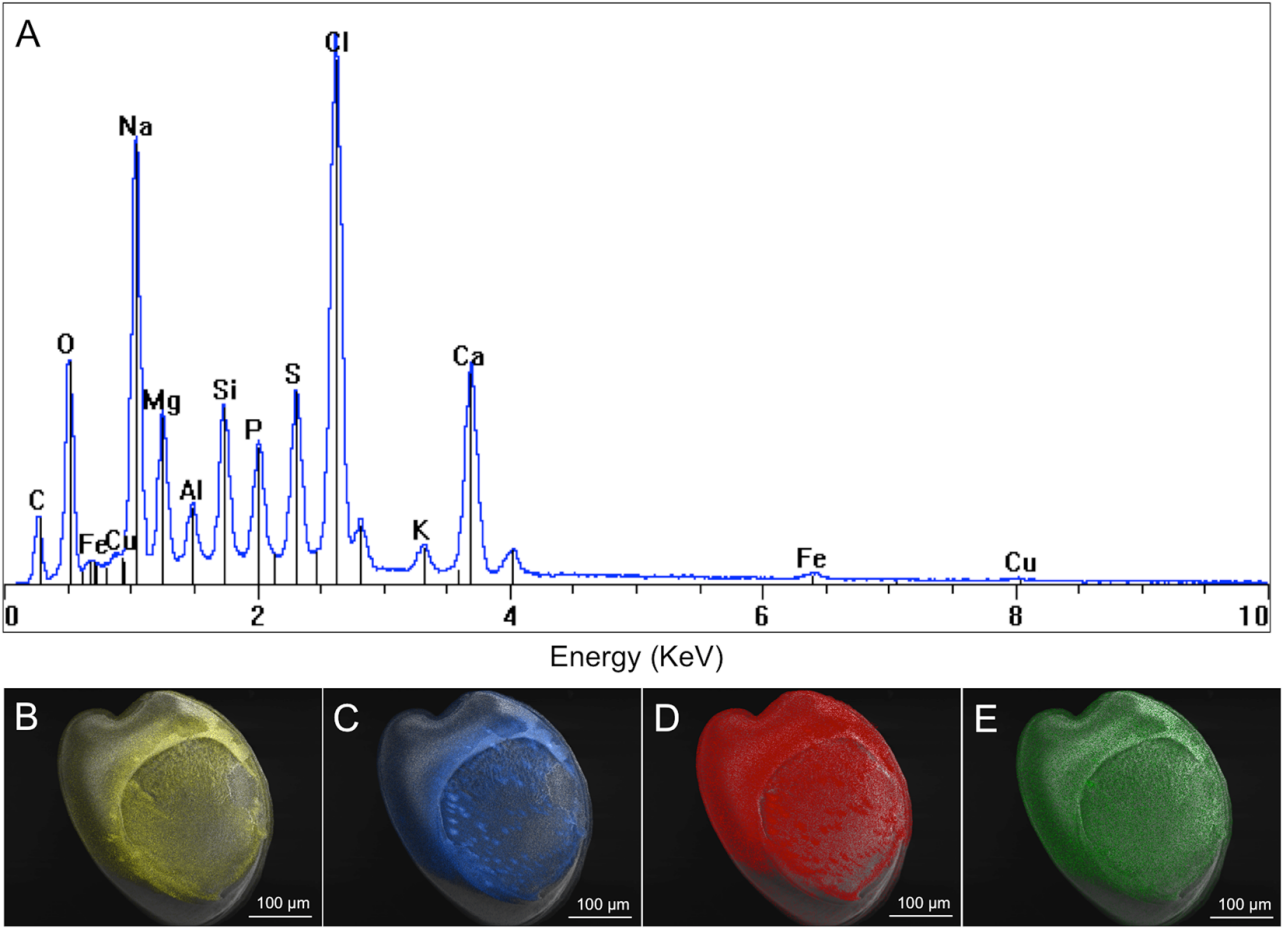
(**A**) Energy Dispersive X-ray spectroscopy (EDS) spectra of the adhesive footprint of *Crassostrea gigas* settled larvae. (**B**–**E**) EDS mapping of (**B**) sulfur, (**C**) chloride, (**D**) sodium, (**E**) potassium, in returned larvae after settlement.

Energy Dispersive X-ray spectroscopy (EDS) revealed the elemental composition of the adhesive footprint. In Fig. 5A, an EDS spectrum of upside-down larvae with adhesive that had the same orientation to the EDS detector presents carbon, oxygen, iron, copper, sodium, magnesium, aluminium, silicon, phosphorus, sulfur, chloride, potassium and calcium peaks. Iron, copper, magnesium, aluminium and phosphorus were not precisely located with spatial mapping analysis, probably because of their low abundance in the sample. Silicon was detected in small dots in the adhesive, potentially resulting from debris of diatom frustules. Sodium, chloride and potassium are co-located in aggregates in the adhesive (Fig. 5C–E). EDS spatial mapping located nitrogen in the adhesive, not annotated in the spectra because of its detection between the carbon and oxygen peaks. Sulfur is also specifically present in the adhesive disc (Fig. 5B).

**Figure 6:**
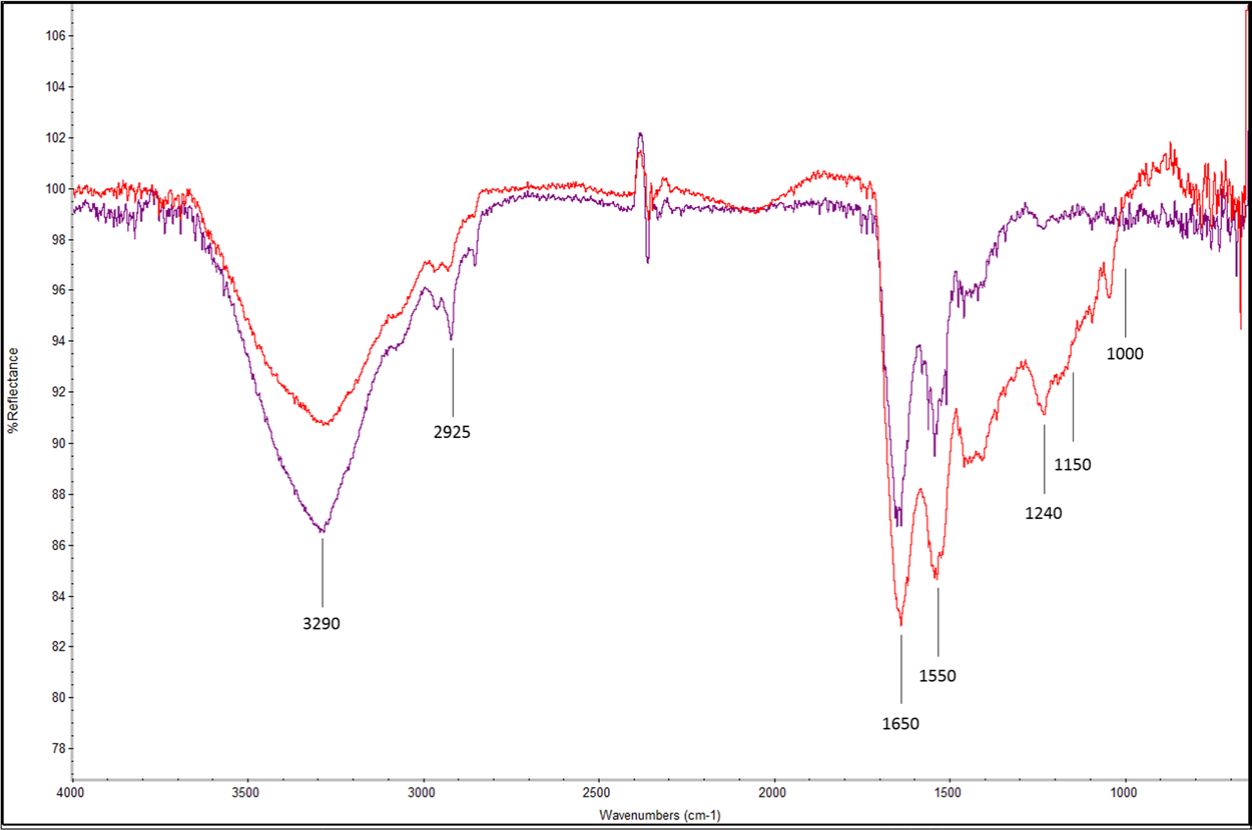
Fourier Transform Infrared Spectra of the adhesive footprint of *Crassostrea gigas* larvae in reflection mode. Red: inner zone of adhesive footprint. Blue: outer zone of adhesive footprint.

The FTIR analysis did not show differences between the inner (in red) and outer (in blue) zones of the adhesive footprint (Fig. 6). Peak intensity difference in spectra was explained by the difference in adhesive thickness between outer and inner zones. A clear protein signal was obtained from the adhesive footprint of *C. gigas* with dominant amide I and II bands near 1,650 and 1,550 cm^−1^. The peak at 3,290 cm^−1^ corresponds to the amide N-H stretch and/or C=O bounds. Phosphorylated proteins could be present between 900 and 1,200 cm^−1^. Polysaccharides can also be seen at 1,000–1,150 cm^−1^. Potential sulfated polysaccharides are present with a band in the region between 1,240 and 1,245 cm^−1^. The band observed at 2,925 cm^−1^ can be assigned to C-H stretching.

Five protein extraction protocols were tested on adhesive foot print (see Supplementary Note) based on chaotropic agent (urea, or guanidine hydrochloride acid, or NaOH), and/or chelating agent (EDTA). No adhesive fibres solubilisation was observed in microscopy observation and protein assay after these treatments.

Enzymatic digestion of the adhesive footprint from *C. gigas* was analysed by Nanoscale liquid chromatography coupled to tandem mass spectrometry (nanoLC MS/MS). Forty-two proteins were identified in all three replicate samples (see Supplementary Table 1). All proteins detected in each replicate are listed in Supplementary Table 2 and were successfully assigned to an oyster genomic sequence. Molecular function annotation showed that 82% of the 42 proteins mutually identified had a binding domain, 43% were annotated for catalytic activity and 20% had structural molecular activity. Expression profiles of the genes encoding these proteins were analysed based on the transcriptomic data from Zhang *et al*. (2012) (see Supplementary Table 1). All the genes corresponding to the detected proteins are expressed at the pediveliger stage. Interestingly, one gene, the “PREDICTED Uncharacterized protein LOC105327706”, is specifically strongly expressed at the pediveliger stage. This predicted protein was detected by one (7.14% coverage) or two peptides (9.14% coverage) in the three replicates using nanoLC MS/MS analysis, with a MASCOT score of 102.01 ± 6.38 (see Supplementary Table 2, protein sequence in Supplementary Note). This gene was selected for deeper gene expression location by mRNA *in situ* hybridization (ISH).

### mRNA location of “PREDICTED: Uncharacterized protein LOC105327706”

**Figure 7:**
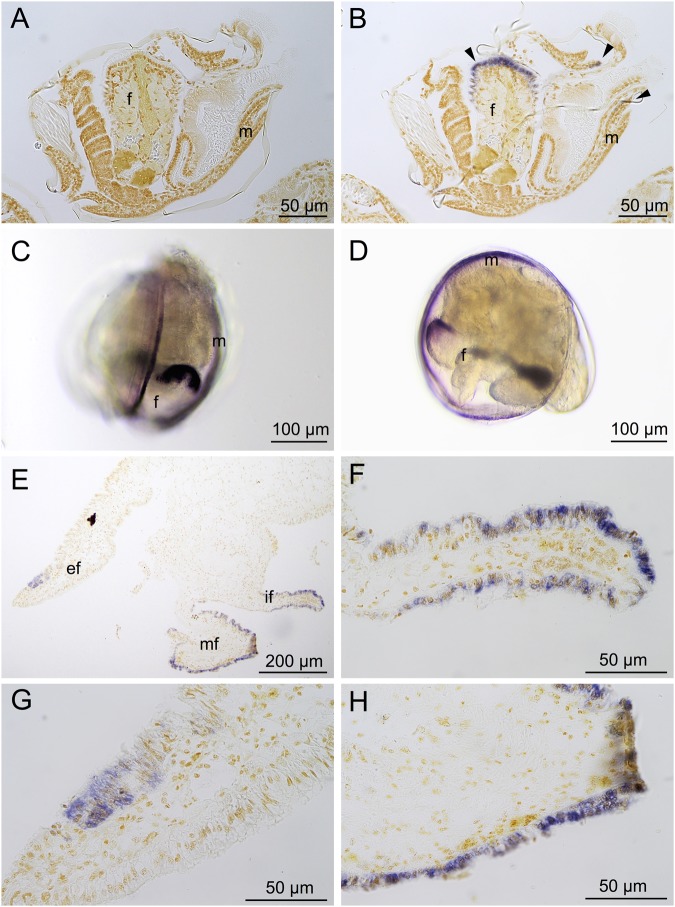
mRNA *in situ* hybridization of *Crassostrea gigas foot mantle protein* (*Cg_fmp*) in pediveliger larvae (**A**–**D**) and adults (**E**–**F**) in histological sections (**A**,**B** and **E**–**H**) and whole mounts (**C**,**D**). A: sense riboprobe hybridization (negative control). (**B**–**H**) Anti-sense riboprobe, blue coloration indicates the positive detection of *Cg_fmp* mRNA. (**C**) Ventral view of a pediveliger larva. (**D**) Left view of a pediveliger larva. (**E**) Adult mantle margin. (**F**) Inner fold of the mantle margin. (**G**) External fold of the mantle margin. (**H**) Middle fold of the mantle margin. f: foot, m: mantle, ef: external fold, mf: median fold, if: inner fold.

The tissue expression pattern of the “PREDICTED: uncharacterized protein LOC105327706” (CGI_10007887) gene was successfully located by whole ISH (WISH) in pediveliger larvae and by ISH in adult specimens of *C. gigas*. According to the tissue expression pattern, we named this gene *Cg_fmp* for *Crassostrea gigas foot mantle protein*. No coloration was observed in the negative control, indicating the specificity of ISH and WISH staining (Fig. 7A). At the pediveliger stage, the *Cg_fmp* mRNA are specifically located in the mantle margin and in the tip of the foot (Fig. 7B–D). In the mantle margin of larvae, only a few rows of epithelial cells are stained (Fig. 7B–D). The tip of the foot appears strongly stained (Fig. 7B–D), as for the mantle, with staining affecting 3 or 4 rows of epithelial cells with a decreasing intensity of staining from the external to the internal zone of the foot. In adults, *Cg_fmp* mRNA are only located in the three folds of the mantle margin (Fig. 7E), more precisely in the cylindrical cells of the external and inner fold (Fig. 7F,G) and in the cubic cells of the median fold (Fig. 7H).

## Discussion

The aim of our study was to make the first exploration of the adhesive of pediveliger *Crassostrea gigas* larvae. Fifty years ago, a first precise description of adhesion and foot structure in the *Ostreidae* was published by H.J. Cranfield for *Ostrea edulis*^24,25^. These founding results^11^ were extended by further histological descriptions of pediveliger larvae in the American oyster *Crassostrea virginica*^26,27^. More recently, the molecular character of several marine biological adhesives has been totally or partially decrypted driven by the biotechnological potential of these molecules^12^. Despite these efforts, no marine larval adhesives have been fully characterised, partly due to the difficulty in obtaining enough biological material to this. Concerning *C. gigas* larvae, no information was yet available concerning structural, chemical and molecular composition of the adhesive despite the economic and ecological importance of this species.

Our observations of *C. gigas* larval settlement behaviour led to similar conclusions to those reported by Cranfield *in O. edulis*^24^. Before cementing, *C. gigas* pediveliger larvae stop swimming, sink, and explore the substrate for a few minutes by sequences of foot contractions. In *O. edulis*, glands presenting a polysaccharide or glycoprotein content, also detected in *C. gigas* by multichrome staining (A, B and D), had a role during the crawling phase^21,25^. Larvae sampled during this crawling phase present cilia overwriting at the extremity of their foot resulting from foot pressing during locomotion. Vesicles observed at the base of the cilia at the tip of the foot probably from gland B, secrete an adhesive substance which subsequently allows the larvae to stick to their substrate during crawling^25^. At the end of the crawling phase, i.e. just before adhesive secretion, larvae begin anchorage to the substrate, weaving filaments between the foot and the substrate. The origin of the secretion of this filament was not precisely determined, but similar observations in *O. edulis* led to the hypothesis that byssus filaments are secreted to the byssus duct by group of glands D^25^. Finally, the foot secretes most of the adhesive by contraction of the principal gland, named “C1” in *O. edulis* or “byssal gland” in *C. virginica*^22,27^. As seen in the present study, this gland is composed of strongly eosinophilic granules and positive picric acid staining observed in histological sections, indicating a proteinaceous composition. After secretion of gland C, adhesive of *C. gigas* presents a fibrous structure, similar to that observed in *O. edulis*^21^ and in *C. virginica*^28^. Our SEM observations revealed an outer and an inner zone with different fibrous structures, suggesting potential differences in composition and complementary roles in their adhesive properties. However, no spectral differences were observed between the two adhesive zones by Fourier Transform InfraRed analysis. Our FTIR investigation made on the *C. gigas* adhesive footprint showed major protein and carbohydrate signals, similarly to the first investigations performed in *O. edulis* by Cranfield in the 70 s with histochemical techniques. The Infrared spectrum of *C. gigas* adhesive is also similar to that of mussel larva adhesive^29,30^. FTIR indicated the molecules and functional group constituting the adhesive, and the presence of proteins could be expected. Indeed, proteins play an essential role in most marine adhesives characterised^12^, particularly in molluscs^5,31^. For example in mussels, proteins with DOPA motifs are directly and essentially implicated in adhesion to other proteins and to the substrate^16,32^. Elemental composition of *C. gigas* larval adhesive determined in this study by energy dispersive X-ray spectroscopy (EDS) indicated the presence of sulfur, as shown in *P. canaliculus*^33^ and *C. virginica* larval adhesive^28^. Sulfur was reported to be implicated in disulfide bonds of adhesive proteins from several marine organisms^34–38^. The presence of calcium, iron, and magnesium in the adhesive may indicate ionic interactions with these elements, which play a key role in some marine bioadhesives^12,39^. Also, the phosphorus detection is in accordance with protein phosphorylation described in some bioadhesive mechanisms^12,40,41^, but the absence of specific localisation during EDS mapping raised doubts about its real involvement in adhesion mechanisms. However, FTIR spectrum does not rule out the presence of phosphoserine vibrational bands in the region from 900–1200 cm^−1^, encouraging further specific study concerning these protein modifications in oyster adhesive^42^. The presence of Mg in the adhesive could indicate complexation with phosphoproteins as observed in sandcastle worm adhesive^43^. Small patches of sodium, chloride and potassium co-localisation in adhesive of *C. gigas* larvae indicated potential sea salt contamination.

The main challenge to investigating the protein composition of oyster larval adhesive by classical proteomic approaches was the very small amount of material produced. No results concerning specific protein extraction are presented in this study because of the tiny amount of material available and the difficulty of solubilizing the adhesive after its secretion and linkage to the substrate (see Supplementary Note). Alternatively, a direct tryptic digestion of adhesive footprints successfully resulted in the identification of 42 protein sequences, potentially implicated in adhesion. A quarter of these proteins have Gene Ontology annotation related to basal cellular functions, like DNA transcription or RNA processing, indicating their nuclear origin: plasminogen activator inhibitor 1 RNA, staphylococcal nuclease domain-containing 1-like, heterogeneous nuclear ribonucleoprotein D-like isoform X2, elongation factor 1-alpha, elongation factor 2, 60S acidic ribosomal P0, 60S ribosomal L5, 40S ribosomal S24 isoform X1 and partial histone H4. These proteins were most probably not from the final adhesive secretion and were not a component of the adhesive footprints. Plasminogen activator inhibitor 1 RNA function in *Crassostrea gigas* is not known, but this protein has a specific domain of nucleotide binding indicating a potential role in RNA regulation or RNA stability. The Staphylococcal nuclease domain-containing 1-like is a coactivator of a transcription factor. Heterogeneous nuclear ribonucleoprotein is also known as a transcription factor. Elongation factor can act as a transcription factor and could be implicated in the regulation of protein maturation. Ribosomal subunits were detected in our sample. Ribosomes are directly implicated in RNA translation for protein synthesis. Histones are involved in chromatin structure. The presence of proteins implicated in DNA transcription and RNA processing indicate high transcription activity at this larval stage. At the pediveliger stage, larvae synthesize the adhesive in the foot gland, which represents a high volume of the larval body. At this stage, this organ is probably the one of the most active in DNA transcription and RNA processing. However, adhesive synthesis is a short term process, because there are only a few days between reaching the pediveliger stage (at 15 days post fertilization at 25 °C) and settlement. Additionally, larval metamorphosis is prepared for during the pediveliger stage and begins a few hours after settlement. During metamorphosis, the foot disappears totally. Proteins implicated in DNA transcription and RNA processing presumably play an important role in the different steps of larval crawling, settlement and metamorphosis.

Interestingly, proteins implicated in protein synthesis and the carbohydrate cycle were also detected: phosphoenolpyruvate carboxykinase-like, disulfide isomerase and disulfide isomerase A3, fructose-bis-phosphate aldolase, aldolase, 14-3-3 zeta/epsilon and peptidyl-prolyl cis trans isomerase-like. Disulfide isomerase, located in the endoplasmic reticulum, catalyses the rearrangement of di-sulfide bonds and interacts with protein glycosylation. This protein was also detected in the proteome of the tube feet adhesive organs of sea urchin^44^. The presence of this enzyme involved in post-translational modifications of proteins is in accordance with sulfur detection in adhesive by EDS analysis. The disulfide bond could play a major role in adhesion mechanisms. Fructose-bis-phosphate aldolase could act as scaffolding protein^45^. 14-3-3 zeta/epsilon are binding proteins involved in transmission pathway or protein assembly. Peptidyl-prolyl cis-trans isomerase binds to and isomerizes specific phosphorylated Ser/Thr-Pro (pSer/Thr-Pro) motifs by inducing conformational changes in a subset of phosphorylated proteins and acts as a molecular switch in multiple cellular processes. The presence of proteins involved in protein synthesis, in relation with the presence of proteins involved in DNA and RNA processing, indicates the high cellular activity of the foot. These proteins are potentially involved in adhesive protein complexation or in adhesive secretion.

Muscular cells constitutive proteins were also detected: filamin A-like isoform X1 and X4, myosin (probably from adductor muscle), actin A3a cytoplasmic, actin alpha-sarcomeric-like isoform X1, paramyosin-like isoform X1, transketolase, intermediate filament, tubulin alpha and beta chain, tropomyosin isoform X6 and gelsolin. Contractions of the foot during crawling phase are made possible by the presence of foot muscles observed in the histological preparations and previously described in oyster pediveliger larvae^11^. Also, proteins involved in cellular energy pathways could come from muscular activity of the foot: fructose-bis-phosphate aldolase, aldolase, arginine kinase, sarcoplasmic calcium binding protein and ATP synthase. Behaviour observations of pediveliger larvae suggest that muscle has an important function during settlement. However, the muscle proteins found in our analysis probably came from cellular contamination of the substrate, and not from the final adhesive.

Among the 42 proteins identified, one predicted uncharacterized protein was detected, which we named *Cg_fmp*. This predicted protein is a sequence of 298 amino acid comprising 20 amino acid that are a peptide signal indicating potential extracellular export or a membrane localization. No conserved domain was detected in this sequence. The 12 amino acid sequence at position 148, which includes 10 glutamates, could lead to an intrinsically disordered region^46^. The region between positions 149 to 181 shows a coiled domain prediction, but this domain does not provide information about the role of *Cg_fmp*. However, an 8 amino acid sequence of consecutive prolines, at position 65, could form polyproline II helix^47,48^. These hydrophobic structures are often involved in protein interactions and protein complex assembly and have been described in terrestrial and marine bioadhesive proteins^12^. Also, the predictive pI of 4.78 of the *Cg_fmp* protein indicates a high negative charge density and “hard” metal-binding ligands. Divalent metal cation (esp. calcium) binding sites have been reported in adhesive proteins^12,49–51^. It is noteworthy that the mRNA level pattern data from Zhang *et al*.^23^ indicates that this gene is strongly expressed at the pediveliger stage. Localization of the expression of the *Cg_fmp* gene in the epithelial cells of the tip of the foot of *C. gigas* pediveliger larvae indicates a potentially close relationship between the biological role of *Cg_fmp* protein and the crawling phase. However, the expression of the *Cg_fmp* gene in larval and adult mantle showed that activity of the gene was more diversified. Even though the localization in mantle epithelial cells is the same for larvae and adults, alternative splicing of *Cg_fmp* RNA could be different between these stages, resulting in splice variants with different physiological properties^52^. Although the precise role of the *Cg_fmp* could not be assessed using mRNA *in situ* hybridization, some hypotheses could be formulated:

(i) *Cg_fmp* protein could be localized in the membrane of epithelial cells, participating in the transmission of external stimuli to nerve cells.
(ii) In addition, *Cg_fmp* could be involved in temporary adhesive composition, or secretion by vesicle trafficking.

The tip of the foot probably has a sensitive function; nerves are present from the base of the foot with pedal ganglia reaching to the tip of the foot with neuronal extensions. During crawling, the cilia overwriting at the tip of the foot of *C. gigas* larvae indicates a close relation between this zone and the substrate. Crawling is essential because it allows oyster larvae to choose a habitat for their whole lives. In addition, settlement is closely mediated by neurotransmitters^53,54^, which could be detected by *Cg_fmp* as sensitive receptor. Detection of the *Cg_fmp* transcript in the mantle also supports the hypothesis that this protein has a sensitive function. The mantle presents cells and muscles mediating specific behaviour in response to environmental stimuli^11,26,55^.

However, the localization of *Cg_fmp* expression in the mantle margin of larvae and adults and in the tip of the larval foot potentially indicates a dual role of the *Cg_fmp* protein. In *O. edulis*, it was shown that the mantle produces final adhesive deposition after foot secretion during larval settlement^21,24^. However, the main role of the oyster mantle is to secrete shell during larval and adult stages^11^. Oyster shell is composed of an inorganic and an organic part, the periostracum. *Cg_fmp* protein could have an adhesive function in the latter at the larval stage. However, the epidermis cells stained in the adult mantle are not those known to secrete the periostracum. Cells stained in the outer mantle margin link the mantle to the shell. Also, cells stained in the middle and inner fold could have adhesive properties. Oysters could modulate water flow by closing the pallial curtain with the middle and inner mantle folds^55^.

Adhesive properties of *Cg_fmp* protein are also supported in crawling behaviour. Crawling is characterized by temporary attachment of the foot mediated by temporary adhesive secretion at the tip of the foot. These secretions were described by Cranfield in *O. edulis*, with the presence of glands opening at the tip of the foot (gland B)^25^. The vesicles observed by SEM at the base of the cilia of the foot tip in *C. gigas* probably result from secretion by this gland. The hypothesis of temporary adhesive secretion by vesicle liberation during the crawling phase implies the role of vesicle mediated transport, and secretion mechanisms with a membrane docking protein. Structural proteins, like intermediate filaments and proteins involved in protein post-translational modification such as peptidyl-prolyl-cis-trans-isomerase, could be related to it. Studies on temporary marine adhesive were performed in echinoderms^44,56–60^. A similar proteomic approach was used on urchin *Parocentrus lividus* adhesive footprints, showing cellular and structural proteins from muscle, and proteins from DNA and RNA processing, like histones^44,59^. Temporary adhesion implies the loss of cells and epidermis fragments on the substrate during detachment. SEM observations of crawling larvae revealed that the strongly ciliated foot of *C. gigas* shows cilia overwriting because of foot pressure on the substrate during crawling. In mussels, cilia play a key role in environmental exploration and byssus formation^61^. Reversible adhesion of the foot probably results in the loss of cilia and epidermis fragments on the substrate during crawling. Additionally, valve contractions observed at the final adhesive release could scrape cilia from the foot and distribute the debris in the final adhesive. In *O. edulis* adhesive footprints, transmission electron microscopy observation indicated the presence of cilia in the final adhesive secretion^21^. This hypothesis is supported by our detection of four proteins presenting a potential role in cyclogenesis (microtubule-associated-like 2 isoform X3, dynein, filamin A-like isoform X1 and X4). Furthermore, proteins implicated in cellular energy could also result from the foot cilia activity.

Our study is the first time a proteomic approach has been successfully performed on *C. gigas* adhesive, revealing 42 proteins potentially implicated in adhesion. The transcript of one uncharacterized proteins, named *Cg_fmp* for *Crassostrea gigas foot mantle protein*, was successfully located in the tip of the foot and in the mantle of pediveliger larvae, probably indicating its role in crawling, as well as in adult mantle, indicating a physiological role at the adult stage. The precise role of this protein needs to be investigated in greater depth using functional molecular approaches. Gene Ontology of the 42 detected proteins and cellular location of the *Cg_fmp* mRNA indicate that these proteins came from oyster cells lost during crawling and probably not from the final adhesive structure. A probable explanation for the detection of cellular proteins in the adhesive analysis is the method employed. This analysis was performed on the total substrate after removal of larvae, and the adhesive footprint seems to remain insensitive to trypsin digestion. Binocular observation of the adhesive footprint, before and after digestion, did not reveal any major changes in its shape. However, contrary to other preliminary tests with an aggressive protein extraction technique (see Supplementary Note), this approach permitted, despite the small amount of available material, to detect proteins involved in the crawling phase of *C. gigas* larvae. It is probably not possible to extract proteins from adhesive footprints because of the insolubility of the secreted and complexed material. Further investigation is needed on the composition of larval oyster adhesive. Laser capture microdissection techniques, which would allow samples to be taken of the foot gland containing adhesive precursors, would be an interesting way to characterise larval adhesive. The first knowledge needed will be the molecular composition of larval adhesive and its mechanistic interactions with its environment. These will potentially allow biotechnological applications of this material. Studies on the chemical mechanics of adhesion will also be necessary to fully describe oyster larval adhesion strategies.

## Methods

### Biological material and larval rearing

*Crassostrea gigas* is class *Bivalvia*, and family *Ostreidae*. *C. gigas* larvae were produced at the Ifremer hatchery facilities in Argenton (France) as described in Petton *et al*. (2013)^62^. Larvae were maintained at 25 °C, 34 PSU, pH 8.2 to 8.3, 95 to 100% saturated O_2_ and fed *ad libitum* on a mix of microalgae (*Tisochrysis lutea* and *Chaetoceros sp*. strain CCAP 1010-3). A constant concentration of 1500 μm^3^.μl^−1^ microalgae was maintained at the exit of each 5-L flow-through rearing tank^63^, leading to production of a mean number of 250 000 ready to settle larvae per tank (corresponding to 50 larvae per mL). At 14 days post fertilization, pediveliger larvae were sampled and placed in a settlement tank with micro-ground shell. In order to capture ready-to-settle larvae for further analysis, specific substrates were placed in the settlement tank: frosted glass (for microscopy observation and proteomic analysis) or polished inox 316 L (for FTIR analysis). The readiness to settle of larvae was checked by binocular observation (magnification ×50).

### Scanning electron microscopy and energy dispersive X-ray analysis

Crawling and settled larvae (n = 1000) were anesthetized with 5% MgCl_2_ for 30 min^64^. Samples were fixed in a 1.5% glutaraldehyde in a 0.1 M sodium cacodylate buffer (1.75% w/v of NaCl, pH 7.2) at 4 °C for 2 h. Samples were washed three times in a solution of 0.1 M sodium cacodylate and dehydrated by successive immersion in alcoholic (50-70-90-100%) and alcoholic hexamethyldisilazane (HMDS) (v:v), absolute ethanol:HMDS (3:1), absolute ethanol:HMDS (1:1), absolute ethanol:HMDS (1:3), and pure HMDS. For visualization of the adhesive, one hundred larvae were taken off the substrate using micro-forceps, then turned over and stuck on adhesive carbon tape. Finally, the samples were coated with gold palladium, or with carbon for energy dispersive X-ray analysis. The larvae were then observed by scanning electron microscopy (Hitachi S-3200N).

### Histology

To describe foot anatomy, histological preparations were made of *C. gigas* pediveliger larvae. Crawling larvae (n = 1000) were anesthetized with 5% MgCl_2_ for 30 min^64^. Samples were fixed in a modified Davidson fixative (12% v/v formaldehyde, 15% v/v ethanol and 5% v/v acetic acid for 24 h at 4 °C. Fixed larvae were decalcified with a solution of 3% ascorbic acid for 12 h^65^. The larvae were then dehydrated by successive immersion in an alcoholic series (50–70–90–100%) and claral® and embedded in paraffin. Serial sections of 5 µm were made on a microtome. Histological sections were stained with haematoxylin and eosin to allow visualization of anatomical structures^66^. To visualized proteins and polysaccharides content, sections were also stained with a multichrome technic (Alcian Blue, Periodic Acid–Schiff’s, Haematoxylin and Picric Acid)^67^. Sections were dehydrated with serial baths of alcoholic solutions (50–70–90–100%) and mounted in Eukitt medium. Images were captured with an inverted microscope Zeiss AxioObserver Z1 and a Canon EOS 750D Camera.

### Fourier transform infrared spectroscopy

To assess the chemical composition of the adhesive, a Fourier Transform InfraRed spectroscopy (FTIR) analysis was made. One cm^2^ polished stainless steel 316 L plaques were placed in the settlement tank containing pediveliger larvae. After 48 h, these plaques were removed, rinsed with distilled water and dried. Adhered larvae were removed from the plaques with micro-forceps under a binocular microscope. All spectra were recorded by an FTIR microscope used in reflection mode. The FTIR microscopy measurements were performed with a Thermo IS50 infrared spectrometer coupled with an infrared Thermo Continuum microscope with a 32x IR objective and a Mercury Cadmium Telluride (MCT) single element detector cooled with liquid nitrogen. Each spectrum was obtained by 65 scans recorded at a resolution of 4 cm^−1^.

### Tryptic digestion and MALDI-TOF/TOF analyses

Freeze-dried adhered larvae were removed from the frosted glass and the maximum contaminant material (faeces, algal deposits) was scraped off with micro-forceps under a binocular microscope. Before analysis, any attached larvae and debris were meticulously removed from the substrate to prevent cellular contamination of sample. One cm^2^ of frosted glass coated with around 300 adhesive footprints was digested with 20 µg of trypsin in 3 mL of 100 mM triethylammonium bicarbonate buffer at 37 °C under gentle agitation for 24 h. Supernatant was transferred into microtubes, evaporated and conserved at −80 °C until analysis. Digestion was repeated for three different glass pieces’.

Samples were re-suspended in TFA 0.1% before nano-LC fractionation. The chromatography step was performed on a nano-LC system (Prominence, Shimadzu). Peptides were concentrated on a Zorbax® 5 × 0.3 mm C18 precolumn (Agilent) and separated on a Zorbax 150 × 75 µm C18 column (Agilent). Mobile phases consisted of 0.1% trifluoroacetic acid, 99.9% water (v/v) (A) and 0.1% trifluoroacetic acid in 99.9% ACN (v/v) (B). The nanoflow rate was set at 300 nl.min^−1^, and the gradient profile was as follows: constant 2% B for 5 min, from 2 to 5% B in 1 min, from 5 to 32% B in 144 min, from 32 to 70% B in 10 min, from 70 to 90% B in 5 min and a return to 2% B in 10 min. The 300 nl.min^−1^ volume of the peptide solution was mixed with 1.2 µL.min^−1^ volumes of solutions of 5 mg.ml^−1^ CHCA matrix prepared in a diluted solution of 50% ACN with 0.1% TFA. Twenty second fractions were spotted with an AccuSpot spotter (Shimadzu) on a stainless steel Opti-TOF™ 384 target.

MS experiments were carried out on an AB Sciex 5800 proteomics analyser equipped with TOF/TOF ion optics and an OptiBeam™ on-axis laser irradiation with 1000 Hz repetition rate. The system was calibrated immediately before analysis with a mixture of des-Arg-Bradykinin, Angiotensin I, Glu1-Fibrinopeptide B, ACTH (18–39), ACTH (7–38), and mass precision was greater than 50 ppm. All acquisitions were made in automatic mode. A laser intensity of 3400 was typically employed for ionizing. MS spectra were acquired in positive reflector mode by combining 1000 single spectra (5 × 200) in the mass range from 700 to 4000 Da. MS/MS spectra were acquired in the positive MS/MS reflector mode by combining a maximum of 2500 single spectra (10 × 250) with a laser intensity of 4300. For the tandem MS experiments, the acceleration voltage applied was 1 kV and air was used as the collision gas, with gas pressure set to medium.

The fragmentation pattern was used to determine the sequence of the peptide. Database searching was performed using the Mascot 2.5.1 program (Matrix Science) on the dedicated database of *C. gigas* (28 028 proteins). The variable modifications allowed were as follows: C-Carbamidomethyl, K-acetylation, methionine oxidation, and dioxidation. “Trypsin” was selected and two miscleavages were also allowed. Mass accuracy was set to 200 ppm and 0.6 Da for MS and MS/MS mode, respectively. Common sequences from three replicates were isolated and identified. Functional annotation was performed with BLAST2GO 4.0.7 software. For each sequence, the RNA expression profile was visualized with Supplementary Table S14 from the oyster genome publication by Zhang *et al*.^23^. For each gene sequence, the number of Reads Per Kilobase Million (RPKM) was plotted by development stage.

### mRNA *in situ* hybridization

One of the proteins identified by the proteomic approach was selected as a candidate for mRNA *in situ* hybridization (ISH) to locate the tissue expression of the gene encoding this protein. One fragment of 596 bp from position 162 to position 757 of the cDNA (XM_011428314.1 PREDICTED: *Crassostrea gigas* uncharacterized LOC105327706 (LOC105327706), mRNA) was amplified by RT-PCR using total RNA previously extracted from pediveliger *C. gigas* with TRI-Reagent® according to manufacturer’s instructions. Riboprobe synthesis was performed as described by Fabioux *et al*.^68^. The amplified fragment of cDNA was sub-cloned in a pCR4-TOPO plasmid, transformed in *E. coli* TOP10 bacteria and purified. After *in vitro* transcription of linearized plasmid, sense and antisense DIG-tagged ssRNA probes were purified. Hybridization with sense riboprobe was used as a negative control.

Histological preparation of pediveliger larvae was performed according to the protocol previously described for histological coloration. ISH on histological preparations was made according to Boullot *et al*.^69^. The whole mount ISH (WISH) protocol was performed according to Nederbragt *et al*.^70^ and Jackson *et al*.^71^. Hybridization was performed with 500 pg.µL^−1^ of labelled riboprobe at 65 °C. The sample were stained with anti-digoxigenin antibody coupled to alkaline phosphatase. Revelation was made with NBT (nitro blue tetrazolium) and BCIP (5-bromo-4-chloro-3-indolylphosphate) in the dark at 35 °C. Images were captured with an inverted microscope Zeiss AxioObserver Z1 and a Canon EOS 750D Camera.

## Electronic supplementary material

Supplementary Table 2

Supplementary Note

Supplementary Video 1
