# Author Correction: Proteinaceous secretion of bioadhesive produced during crawling and settlement of *Crassostrea gigas* larvae

**DOI:** 10.1038/s41598-020-60487-4

**Published:** 2020-04-14

**Authors:** Valentin Foulon, Sébastien Artigaud, Manon Buscaglia, Benoit Bernay, Caroline Fabioux, Bruno Petton, Philippe Elies, Kada Boukerma, Claire Hellio, Fabienne Guérard, Pierre Boudry

**Affiliations:** 1 Laboratoire des Sciences de l’Environnement Marin (LEMAR), UMR 6539 CNRS/UBO/IRD/Ifremer, Institut Universitaire Européen de la Mer, Technopole Brest-Iroise, Rue Dumont d’Urville, 29280 Plouzané́, France; 2 Plateforme Proteogen SF ICORE, Université de Caen Basse-Normandie, 14032 Caen Cedex, France; 3 Ifremer, Laboratoire des Sciences de l’Environnement Marin, UMR 6539 CNRS/UBO/IRD/Ifremer, Centre Bretagne, 29280 Plouzané, France; 4 Plateforme d’Imagerie et de Mesures en Microscopie, Université de Bretagne Occidentale, 29200 Brest, France; 5 Ifremer, Laboratoire Détection, Capteurs et Mesures (LDCM), Centre Bretagne, 29280 Plouzané, France

Correction to: *Scientific Reports* 10.1038/s41598-018-33720-4, published online 17 October 2018

A supplementary file containing Supplementary Video 1 was omitted from the original version of this Article. This has been corrected in the HTML version of the Article; the PDF version was correct at time of publication.

