# Supplementary material for: Proteinaceous secretion of bioadhesive produced during crawling and settlement of Crassostrea gigas larvae
Source: Sci Rep. 2018 Oct 17;8:15298. doi: 10.1038/s41598-018-33720-4 (PMC6193008; doi:10.1038/s41598-018-33720-4)
Supplement: Supplementary file 2 — Supplementary Note [file 41598_2018_33720_MOESM2_ESM.pdf]

Proteinaceous secretion of bioadhesive produced during crawling and settlement of *Crassostrea gigas* larvae.

Valentin Foulon*[1], Sébastien Artigaud[1], Manon Buscaglia[1], Benoit Bernay[2], Caroline Fabioux[1], Bruno Petton[3], Philippe Elies[4], Kada Boukerma[5], Claire Hellio[1], Fabienne Guérard[1], Pierre Boudry[3].

[1]Laboratoire des Sciences de l'Environnement Marin (LEMAR), UMR 6539 CNRS/UBO/IRD/Ifremer, Institut Universitaire Européen de la Mer, Technopole Brest-Iroise, Rue Dumont d'Urville, 29280 Plouzané, France

[2] Plateforme Proteogen SF ICORE, Université de Caen Basse-Normandie, 14032 Caen Cedex, France

[3] Ifremer, Laboratoire des Sciences de l'Environnement Marin, UMR 6539 CNRS/UBO/IRD/Ifremer, Centre Bretagne, 29280 Plouzané, France

[4] Plateforme d'Imagerie et de Mesures en Microscopie, Université de Bretagne Occidentale, 29200 Brest, France

[5] Ifremer, Laboratoire Détection, Capteurs et Mesures (LDCM), Centre Bretagne, 29280 Plouzané, France

*Correspondence to valentin.foulon@univ-brest.fr

Supplementary Note 1. Protein extraction buffer tested in 1000 adhesive footprint of *Crassostrea gigas* larvae (n=5). For all buffers, after extraction, and sample concentration, no proteins were detected by Bradford assay, and no macroscopically changes in binocular observation were present. These methods were not retained for proteomic approach.

Extraction Buffer	Urea 8M<br>SDS 2%<br>Triton x-100 2%<br>CHAPS 2%	NaOH 0,1 M<br>EDTA 0,05 M<br>SDS 2%<br>β-mercaptoethanol 2%	Tris-HCl 1,5 M<br>pH 8,5<br>Guanidine hydrochloride 6M<br>EDTA 0,02M<br>DTT 0,5 M	Guanidine hydrochloride 2 M<br>Hydroxylamine hydrochloride 2M<br>K_2CO_3 0,2 M<br>pH 9	Tris-HCl 0,6M<br>pH 6,8<br>SDS 2%<br>DTT 0,5M
Extraction condition	1h 60°C	30min 90°C	1h 60°C	4h 45°C	3h 60°C
Binocular observation	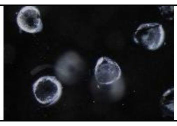	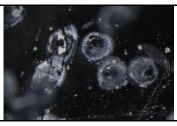	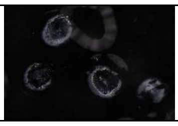	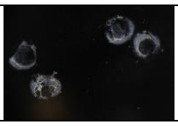	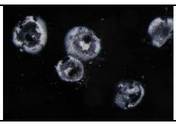
Reference	Homelab protocol	Von Der Haar T. *et al.* Optimized protein extraction for quantitative proteomics of yeasts. *PloS one*, *2*(10), e1078 (2007).	Kamino K. *et al*. Barnacle cement proteins importance of disulfide bonds in their insolubility. *Journal of Biological Chemistry*, *275*(35), 27360-27365 (2000).	Tan YP. *et al*. Infiltration of chitin by protein coacervates defines the squid beak mechanical gradient. *Nature chemical biology*, *11*(7), 488 (2015).	Santos R. *et al*. First insights into the biochemistry of tube foot adhesive from the sea urchin *Paracentrotus lividus* (Echinoidea, Echinodermata). *Marine biotechnology*, *11*(6), 686 (2009).

Supplementary Table 1. Protein list detected in adhesive footprint of *Crassostrea gigas* larvae. For each sequence, Gene Ontology for molecular function (GO F), cellular component (GO C), and biological process (GO P) were annotated. Gene expression (RPKM) by stage was represented with data from Zhang et al. 2012. Larval stage: Eggs (E), Cleavage (C), Embryo (Em), Trochophore (T), D-shape larvae (D), Umbo (U), Pediveliger (P). Spat (S). Juvenile (J). Adult (Ad). For adult stage, 11 different organs were represented from the left to the right: outer mantle margin, inner mantle margin, digestive gland, female gonad, gill, adductor muscle, hemolymph, labial palp, remaining tissues, female gonad, and male gonad.

**Gene ID**	**Protein name**	**GO F**	**GO C**	**GO P**	**Gene expression (RPKM) profile by stage (E-U: Eggs to larvae, P: pediveliger, S-J: after metamorphosis, Ad: Adult)**
CGI_10017112	Actin	ATP binding; structural constituent of cytoskeleton	striated muscle thin filament; membrane raft	hermaphrodite genitalia development; nematode larval development; embryo development ending in birth or egg hatching; apoptotic process; cytokinesis; actin cytoskeleton organization; inductive cell migration	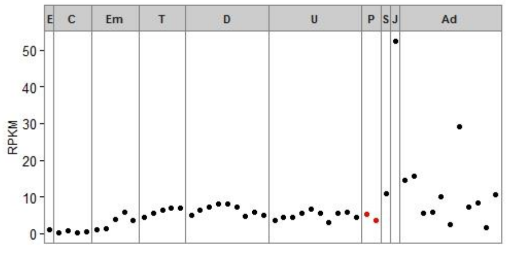
CGI_10003110	Actin alpha - sarcomeric-like isoform X1	actin filament binding; calcium ion binding		actin crosslink formation; actin filament bundle assembly	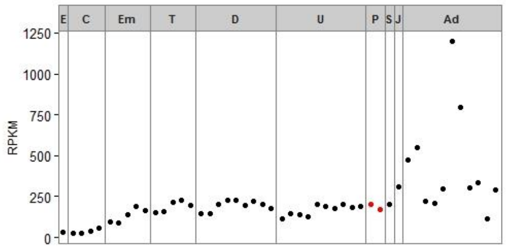
CGI_10003491	Actin A3a cytoplasmic	ATP binding			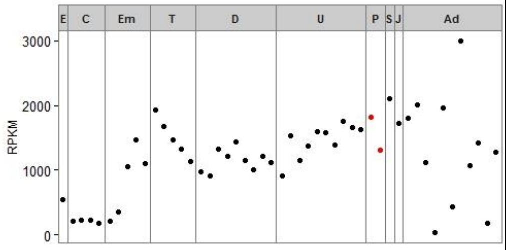
CGI_10018876	Actin adductor muscle	ATP binding	cytoskeleton; cytoplasm		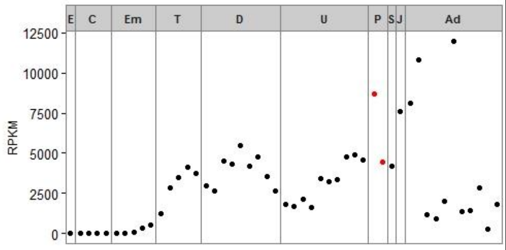
CGI_10024887	Dynein heavy chain axonemal	ATPase activity; ATP binding; microtubule motor activity; actin binding; kinase activity	dynein complex	phosphorylation; microtubule-based movement	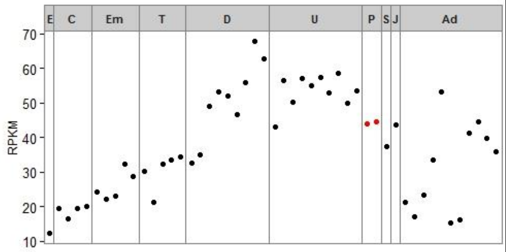

CGI_10005339	echinoderm microtubule-associated -like 2 isoform X3	microtubule binding	integral component of membrane	regulation of microtubule nucleation; negative regulation of microtubule polymerization	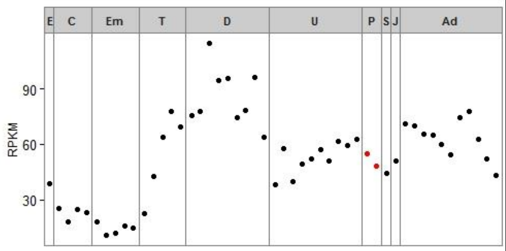
CGI_10003521	filamin-A-like isoform X1	actin filament binding			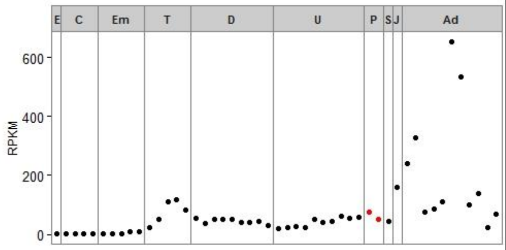
CGI_10012231	filamin-A-like isoform X4	actin filament binding			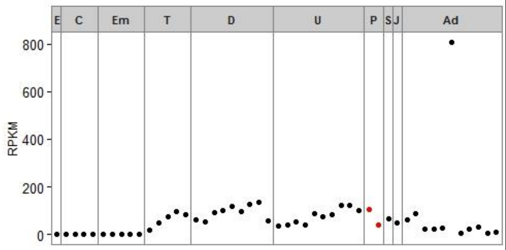
CGI_10002689	gelsolin 2	actin binding; calcium ion binding		actin filament severing; actin filament capping; actin nucleation; actin filament polymerization; actin filament fragmentation	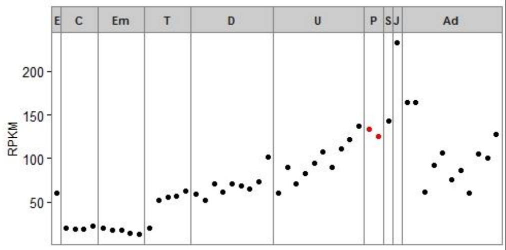
CGI_10010873	Intermediate filament	structural molecule activity	intermediate filament		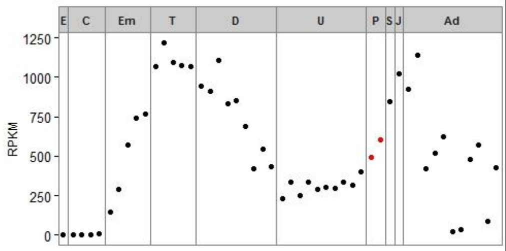
CGI_10022093	Myosin	actin filament binding; ATP binding; calmodulin binding; motor activity	myosin filament; myofibril	metabolic process	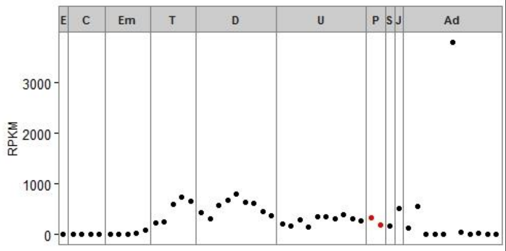
CGI_10025109	Myosin	ATP binding; calmodulin binding; actin binding; motor activity	myosin filament; myofibril	metabolic process	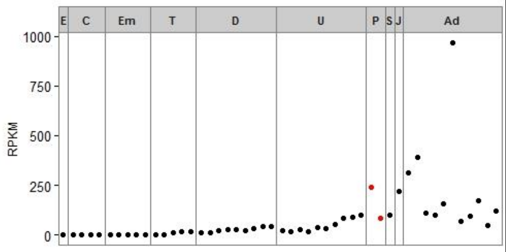

CGI_10001653	paramyosin-like isoform X1	motor activity	myosin filament; myofibril	metabolic process	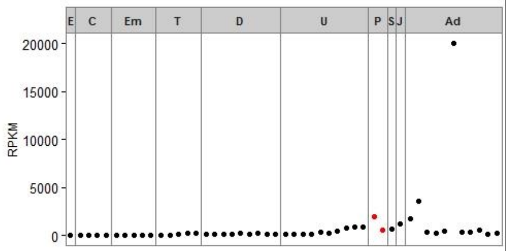
CGI_10006807	Sarcoplasmic calcium-binding	calcium ion binding			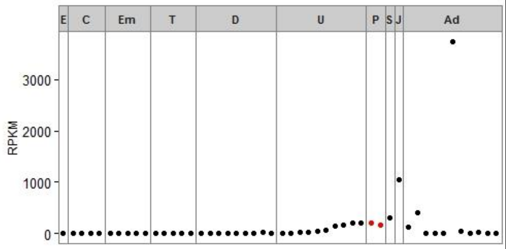
CGI_10024999	tubulin alpha-1A chain-like	GTP binding; GTPase activity; structural constituent of cytoskeleton	cytoplasm; microtubule	metabolic process; microtubule-based process	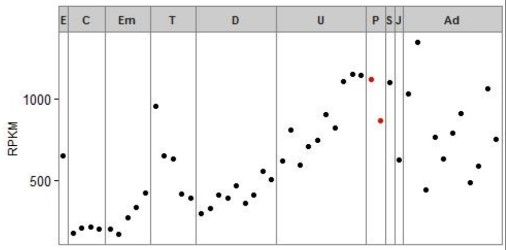
CGI_10012330, CGI_10010163, CGI_10019835	tubulin beta chain	GTP binding; GTPase activity; structural constituent of cytoskeleton	cytoplasm; microtubule	metabolic process; microtubule-based process	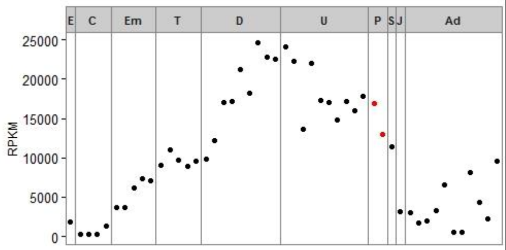
CGI_10028694	Transketolase 2	transketolase activity; metal ion binding		metabolic process	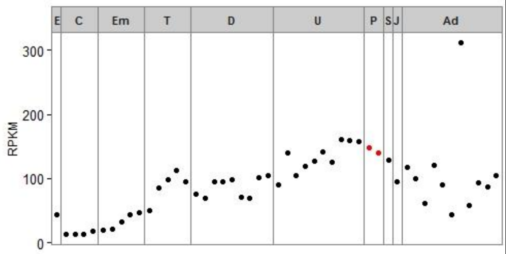
CGI_10013163	tropomyosin isoform X6				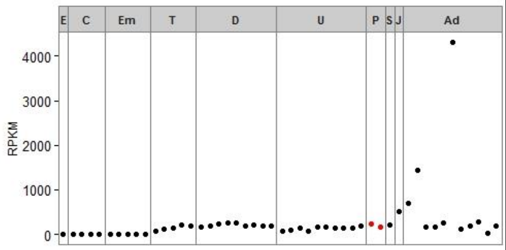
CGI_10025556	Aldolase	fructose-bisphosphate aldolase activity		glycolytic process	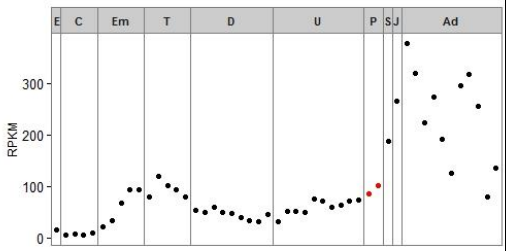

CGI_10013347	ATP synthase subunit mitochondrial	ATP binding; proton-transporting ATP synthase activity, rotational mechanism	proton-transporting ATP synthase complex, catalytic core F(1)	ATP hydrolysis coupled proton transport; ATP synthesis coupled proton transport	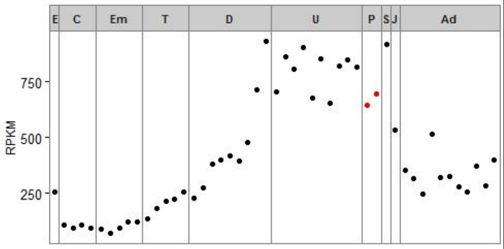
CGI_10019801	Fructose-bisphosphate aldolase	fructose-bisphosphate aldolase activity		glycolytic process	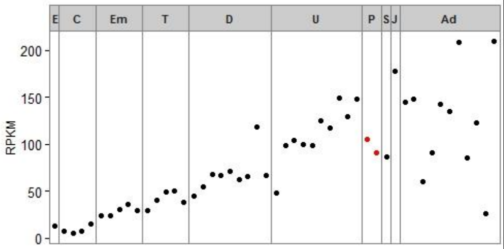
CGI_10014916	phosphoenol pyruvate carboxykinase [GTP]-like	GTP binding; phosphoenol pyruvate carboxykinase (GTP) activity; metal ion binding; kinase activity	mitochondrion	gluconeogenesis; phosphorylation	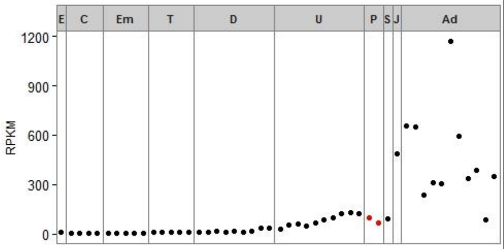
CGI_10012474	elongation factor 1-alpha	GTP binding; translation elongation factor activity; GTPase activity	cytoplasm; integral component of membrane	translational elongation	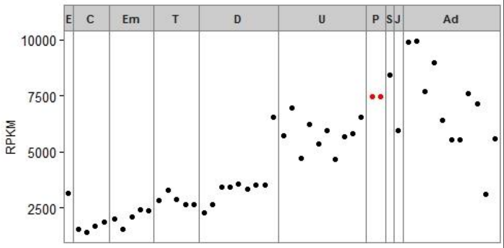
CGI_10001407	elongation factor 2	GTP binding; translation elongation factor activity; GTPase activity	cytoplasm; nematode larval development	hermaphrodite genitalia development; nematode larval development; oogenesis; determination of adult lifespan; embryo development ending in birth or egg hatching; apoptotic process; translational elongation;	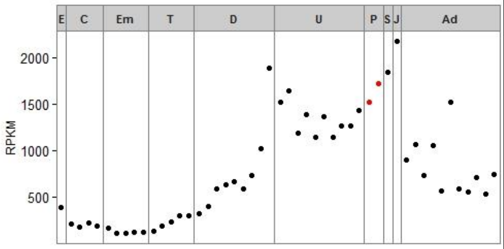
CGI_10028469	heterogeneous nuclear ribonucleo A1-like isoform X1	nucleotide binding; nucleic acid binding	intracellular ribonucleoprotein complex; viral nucleocapsid		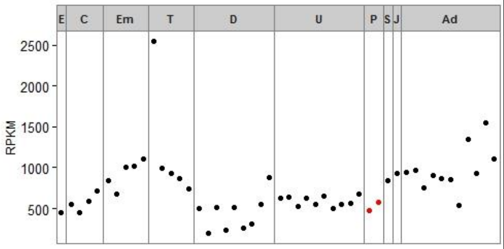
CGI_10020922	heterogeneous nuclear ribonucleo D-like isoform X2	nucleotide binding; nucleic acid binding	intracellular ribonucleoprotein complex; viral nucleocapsid		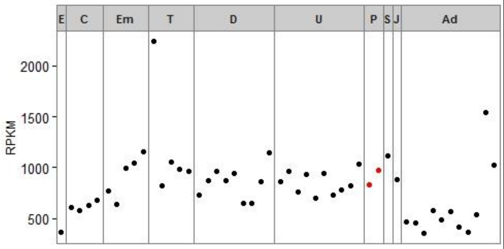

CGI_10017621	heat shock 90	ATP binding; unfolded protein binding		protein folding; response to stress	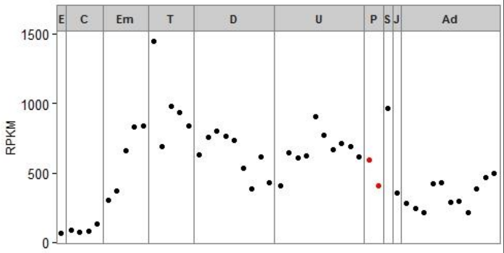
CGI_10008056	histone H4-partial	protein domain specific binding; poly(A) RNA binding; DNA binding; histone binding; protein	nucleosome; nuclear chromosome, telomeric region; nucleoplasm; extracellular exosome	positive regulation of gene expression, epigenetic; cellular protein metabolic process; DNA-templated transcription, initiation; negative regulation of megakaryocyte differentiation; double-strand break repair via	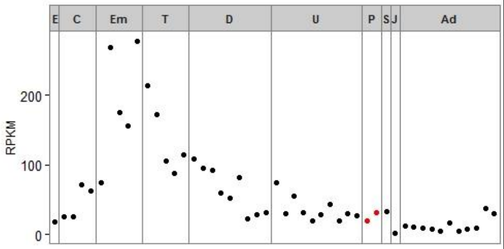
CGI_10028871	Plasminogen activator inhibitor 1 RNA-binding	mRNA 3'-UTR binding			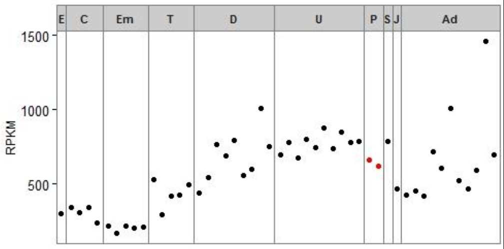
CGI_10001493	40S ribosomal S24 isoform X1	nucleotide binding; structural constituent of ribosome	cytosolic small ribosomal subunit	maturation of SSU-rRNA from tricistronic rRNA transcript (SSU-rRNA, 5.8S rRNA, LSU-rRNA); primitive hemopoiesis; translation; definitive hemopoiesis	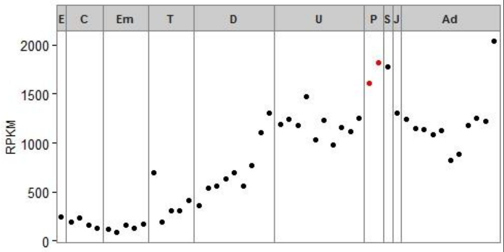
CGI_10027192	60S ribosomal L5	structural constituent of ribosome; 5S rRNA binding	cytosolic large ribosomal subunit	ribosomal large subunit assembly; translation	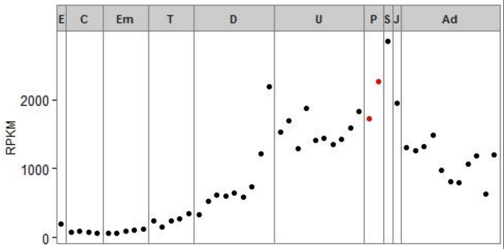
CGI_10010682	60S acidic ribosomal P0	structural constituent of ribosome; large ribosomal subunit rRNA binding	cytosolic large ribosomal subunit; preribosome, large subunit precursor	chordate embryonic development; cytoplasmic translation; ribosome biogenesis	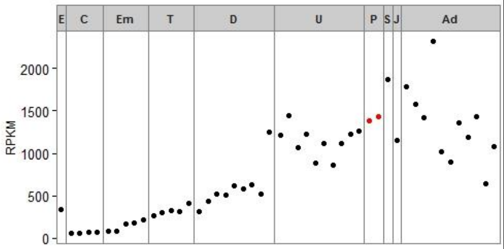
CGI_10026535	staphylococcal nuclease domain-containing 1-like	GTP binding; transcription cofactor activity	membrane; RISC complex	posttranscriptional gene silencing by RNA; small GTPase mediated signal transduction; regulation of nucleic acid-templated transcription	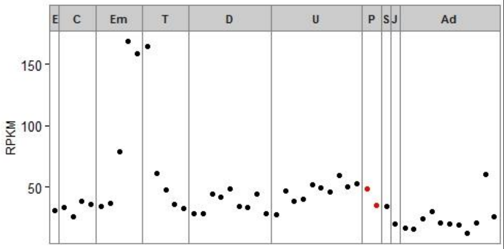

CGI_10024981	mammalian ependymin-related 1-like	calcium ion binding	extracellular exosome	cell-matrix adhesion	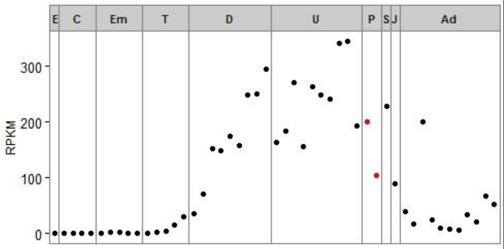
CGI_10021481	Arginine kinase	ATP binding; kinase activity		phosphorylation	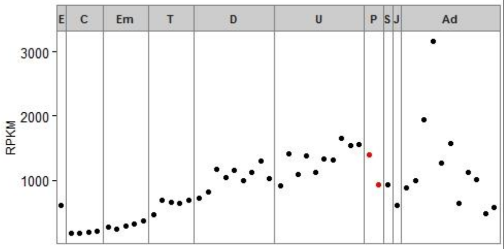
CGI_10026048	disulfide isomerase	dioxygenase activity; protein disulfide isomerase activity; protein disulfide oxidoreductase activity	endoplasmic reticulum	cell redox homeostasis; oxidation-reduction process; protein folding	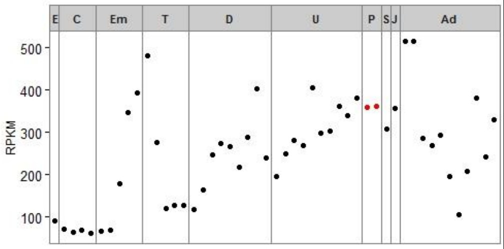
CGI_10011652	disulfide-isomerase A3	protein disulfide isomerase activity	endoplasmic reticulum	metabolic process; cell redox homeostasis; protein folding	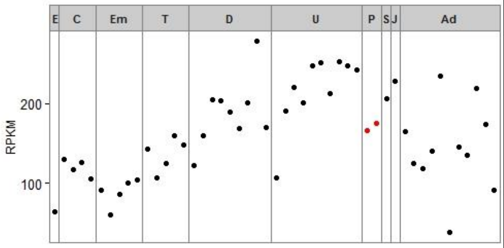
CGI_10025180	peptidyl-prolyl cis-trans isomerase-like	peptidyl-prolyl cis-trans isomerase activity; chemoattractant activity; chemokine activity		response to hypoxia; positive chemotaxis; positive regulation of macrophage chemotaxis; protein peptidyl-prolyl isomerization; protein folding; cell chemotaxis	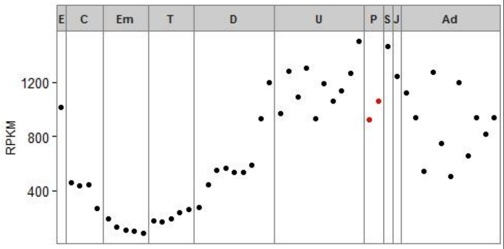
CGI_10015492	78 kDa glucose-regulated	ATP binding	endoplasmic reticulum lumen		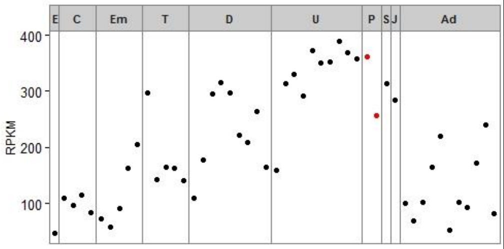
CGI_10024268	14-3-3 epsilon	protein domain specific binding; protein kinase C inhibitor activity; protein heterodimeri	chromosome; microtubule associated complex; plasma membrane; nucleoplasm; centrosome; germline ring canal;	mitotic G2 DNA damage checkpoint; positive regulation of growth; pole cell migration; regulation of mitotic nuclear division; germarium-derived oocyte fate determination; determination of adult	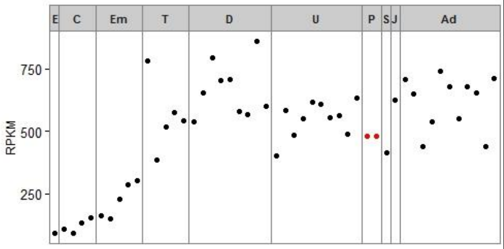

CGI_10014966	14-3-3 zeta isoform X1	protein domain specific binding			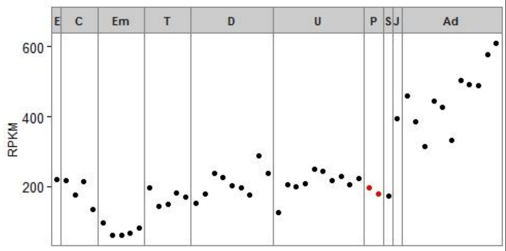
CGI_10007887	PREDICTED: uncharacteriz ed protein LOC10532770 6				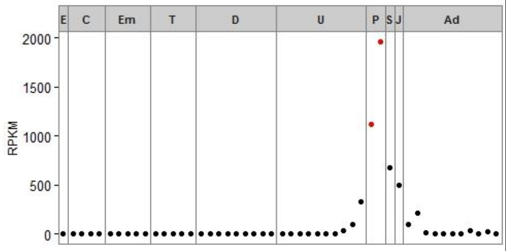

Supplementary Note 2. Protein sequence of the “PREDICTED: uncharacterized protein LOC105327706” detected in the proteomic analysis, Gene ID: CGI_10007887.

```
>gi|762088587|ref|XP_011426616.1| PREDICTED: uncharacterized protein
LOC105327706 [Crassostrea gigas]
MERLVYSVFFLTVCFLISDSSCLDMAAKKFSLFKSRQSFNNFKKRSFSGDNLNVGGKPQVKRQDPPPPPP
PPAVDLWALLGTSRENFIEEHEEGLTTDVMTDIKKQMRDNLNDVKNIAELWARAISCHIFDAMTQAKRTT
GDTDPGIEEEIEEFEEEEEEKDLEELLASFDIDENNLEELVKGLPLPGIVLQCQESPLIMRTGDDTVDKLQ
TLAICGRFLMVKTVEAVDQHDVDTVDNFVDGFERYMKGALTFIHMIFLAVEEPDNPSVREFTRLLQENKL
HELKRYLQSAIAGIKHRK
```
